# An interview with Kevin O’Brien

**DOI:** 10.1590/2177-6709.22.5.018-021.int

**Published:** 2017

**Authors:** Kevin O’Brien

**Affiliations:** » Manchester orthodontic specialty training in 1986. » PhD research into the effectiveness of orthodontic treatment. » Associate Professor in the School of Dental Medicine at the University of Pittsburgh, in 1991. » Professor of Orthodontics in 1996 and Dean of the School of Dentistry at The University of Manchester, from 2004 to 2007. » Associate Dean of the Faculty of Medical and Human Sciences at the University of Manchester, from 2007 to 2010. » Chair of the UK General Dental Council, from 2011 to 2013. » Over 90 scientific papers published, mostly in the AJO-DO. » Gave the Northcroft Memorial and Ballard Lectures for the British Orthodontic Society. » Awarded the Turpin, Dewel, and Jarabak awards by the American Association of Orthodontists. » Currently Director of the Manchester Academic Health Science Centre Clinical Trials Unit.

**Figure:**
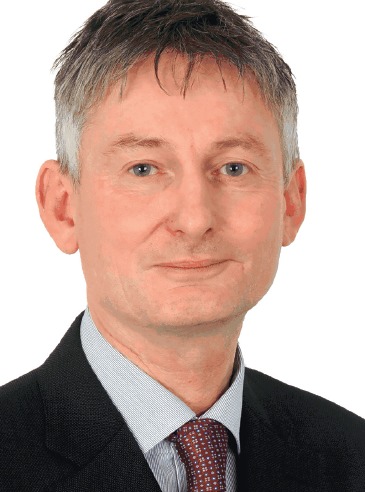

I was delighted to receive the invitation to coordinate this interview with Kevin O’Brien. My first contact with his work occurred in 2009 when I came across two of his papers^1^ ^,^ ^2^ that had changed my mind about research. Since then, his thoughts about the scientific evidence available also influenced several people around the world. His surprisingly successful blog^3^ had reached a remarkable number of 48,000 reads last June. Having as his main research interests the clinical trials of the care of children with severe dentofacial problems, currently, he divides his professional time among the University, Manchester Academic Health Science Centre, his blog and lectures abroad. He spends his free time with his beloved and supportive family: his kind and sweet wife, Janet, his two daughters Jennifer and Claire, his son Jonathan and his lovely grandchildren, Rory and Lois. Besides his family, Kevin loves football (a passionate Manchester United supporter with his name on one of the chairs of the stadium), likes cooking (makes delicious pizzas), looking after his garden and cycling in Manchester. My acknowledgments to Professors José Augusto Mendes Miguel, Jonathan Sandler and Ki Beon Kim, who collaborated in the development of this interview.

Klaus Barretto Lopes - interview coordinator 

Recebi com grande satisfação o convite para coordenar a entrevista com o Prof. Kevin O’Brien. Meu primeiro contato com o trabalho dele ocorreu em 2009, quando me deparei com dois artigos^1^ ^,^ ^2^ que influenciaram para sempre meus conceitos científicos. Desde então, seus pensamentos sobre a evidência científica disponível também têm influenciado muitas pessoas ao redor do mundo. Seu surpreendentemente bem-sucedido *blog* ^3^ atingiu o notável número de 48.000 leituras no último mês de junho. Tendo como principal interesse o tratamento de crianças com problemas dentofaciais severos, atualmente ele divide o seu tempo profissional entre a universidade, a *Manchester Academic Health Science Centre*, o seu *blog* e a realização de palestras mundo afora. O tempo pessoal ele aproveita com a sua acolhedora família, que está sempre por perto e o apoia: sua gentil e querida esposa, Janet; suas duas filhas, Jeniffer e Claire; seu filho, Jonathan; e seus adoráveis netos, Roury e Lois. Além da família, Kevin adora futebol (torcedor apaixonado do Manchester United, tem seu nome em uma das cadeiras do estádio), gosta de cozinhar (faz pizzas deliciosas), cuidar do seu belo jardim e pedalar por Manchester. Meus sinceros agradecimentos aos Profs. José Augusto Mendes Miguel, Jonathan Sandler e Ki Beom Kim, que colaboraram na elaboração dessa entrevista.

Klaus Barretto Lopes - coordenador da entrevista 

## CLINICAL TRIALS AND SYSTEMATIC REVIEWS

Could you tell us what you consider the ten most important clinical trials in the last ten years that have influenced clinical practice the most? Jonathan Sandler

I think that the individual trials are not the important factor in changing practice. The best evidence is derived from systematic reviews, and the really important and high-quality ones are published by the *Cochrane Collaboration* ^4^. There are several orthodontic reviews and these are relevant to clinical practice.

Randomized clinical trials (RCTs) are known to provide the best evidence on a specific question, but its high cost, time needed and other difficulties make this method quite selective. What are the criteria to indicate this type of study and in which situation other methods should be suggested? José Augusto Mendes Miguel

A trial is indicated whenever we want to evaluate the effect of interventions. It is also important to remember that a good properly planned trial will include outcomes that are relevant to patients - for example, self-esteem and the perceptions of treatment. This information cannot be obtained by retrospective studies. As an alternative, the analysis of data that we have already collected on our patients may be helpful, but we need to carefully assess any bias in this information.

Systematic reviews have been published in large scale in the main orthodontic journals. A great part of these papers did not bring significant contributions either because studies published on the topic were of low quality or of very diverse methods, not allowing for comparisons. What criteria should be adopted for systematic reviews and meta-analyses to really contribute for a high level of evidence? José Augusto Mendes Miguel

A good systematic review should only include randomized trials. This will ensure that the review is based on high levels of evidence.

Historically, orthodontic researchers have used surrogate outcomes, such as Angle classification, cephalometric measurements or other factors, that did not bring objective answers about the treatment benefits for the patient. At this time and age, shouldn’t the researchers change their focus to patient-centered questions regarding personal satisfaction, self-esteem or quality of life? José Augusto Mendes Miguel

Yes, we have spent many years collecting and analyzing data that is only relevant to orthodontists. We need to consider patients relevant outcomes in all studies. This would be a major step and add substantially to our knowledge.

We already have several RCTs and systematic reviews in the main topics of orthodontics. Why are clinical practice guidelines still not used in Orthodontics as they are in Medicine? Klaus Barretto Lopes

This is a difficult question. My only suggestion is that perhaps we have not been interested enough in making this step change.

## SOCIAL MEDIA

How might social media both positively and negatively affect the practice of Orthodontics in the 21st Century? How would you advise young orthodontists to get the most from social media to enhance their clinical practice? Jonathan Sandler

The development of social media has been remarkable and it should be very useful to us all. However, the dangers are that it is not controlled and people are making extreme claims about treatment and philosophies. This is a clear negative effect. The positive side is that it is great for sharing information, and we need to make sure that people are sufficiently trained and informed to evaluate the quality of this information.

Your blog is one of the most popular orthodontic blogs in the world. This brings you a great exposition resulting in a great number of “likes” and “dislikes”. How do you manage the positive and negative feedback that you receive? Klaus Barretto Lopes

I am very surprised at the success of my blog. At the end of each day, I look at the comments and approve or not accept them. I do my best to answer the most relevant. I try to accept them all, but sometimes they are so extreme that I cannot publish them. It is a side of the blog that is popular and so it is essential that I try to make it as good as possible.

Dental professionals are increasingly using social media with the sole purpose of self-marketing. On the other hand, your blog shows that the internet can be used as an important tool for discussing ideas and providing information. Tell us about this experience and its reach. José Augusto Mendes Miguel

As I said before, I am surprised at the success of the blog. I initially started it as “something to do”, but now it is a major part of my work. When I started, the first posts were read by about 40 people. Now a post is read by 3-7 thousand readers. In June this year, the posts were read by 48,000 people and this is steadily increasing.

How should we prepare to embrace the technological advancement including big data and artificial intelligence? Ki Beom Kim

This is going to be a great opportunity. But we know very little about the possibilities of this new technology. My real concern is that we will use big data in the same way that we used readily available data to run retrospective studies, that we now recognise are biased. So my advice would be that if we are going to collect data we need to make sure that we collect information on every patient. This includes those who terminate treatment early and whose treatment did not “work out so well”.

## CLASS II

Are there still things to be discovered about the treatment of Class II cases or in your view, do we now have all the answers? Jonathan Sandler

While there is a large amount of evidence-based information, I am not sure if we have all the answers. Orthodontic treatment is always going to be a rapidly moving speciality and there are many new developments that need to be tested. For example, there are many questions about the use of fixed functional appliances and even the use of Class II correctors that are based around aligners.

Class II malocclusion can be successfully treated in different ways. As a result, several orthodontic training programs teach orthodontists to treat Class II malocclusion based on personal experience rather than on the best evidence available. Do you think that it is time to evidence-based orthodontic training? Klaus Barretto Lopes

Yes, all training should be evidence-based, if the evidence is there. In the absence of evidence, we have to rely on clinical experience. However, this approach is somewhat flawed.

How would you suggest to improve the perception of evidence-based orthodontics who don’t believe it? Ki Beom Kim

We simply need to educate people to understand how to interpret the literature critically. This should be done in all training programmes. I have come across programmes that do not cover this aspect of training and it is somewhat disappointing.

What do you think is the most urgent issue in orthodontics and how would you expect the future of Orthodontics? Ki Beom Kim

This is difficult, I think that one of the most interesting research areas now is to investigate methods of making teeth move faster. While there has been some work done on this, it has not been of high quality and we still do not have the answer about the effectiveness of this new treatment. The other work that needs to be done is to move orthodontic research away from measuring the morphological effects of treatment to the analysis of patient values. I am sure that we will find much more useful information if we can make this considerable change.
